# Role of Gut Barrier Function in the Pathogenesis of Nonalcoholic Fatty Liver Disease

**DOI:** 10.1155/2015/287348

**Published:** 2015-04-05

**Authors:** Xin Dai, Bangmao Wang

**Affiliations:** Tianjin Medical University General Hospital, Tianjin 300052, China

## Abstract

Nonalcoholic fatty liver disease (NAFLD) is one of the most common forms of chronic liver disease, and its incidence is increasing year by year. Many efforts have been made to investigate the pathogenesis of this disease. Since 1998 when Marshall proposed the conception of “gut-liver axis,” more and more researchers have paid close attention to the role of gut barrier function in the pathogenesis of NAFLD. The four aspects of gut barrier function, including physical, chemical, biological, and immunological barriers, are interrelated closely and related to NAFLD. In this paper, we present a summary of research findings on the relationship between gut barrier dysfunction and the development of NAFLD, aiming at illustrating the role of gut barrier function in the pathogenesis of this disease.

## 1. Nonalcoholic Fatty Liver Disease

Nonalcoholic fatty liver disease (NAFLD) is one of the most common forms of chronic liver disease throughout the world. It is characterized by liver damage similar to that caused by alcohol but occurs in individuals that do not consume toxic quantities of alcohol. It includes a spectrum of liver diseases extending from simple fatty liver through nonalcoholic steatohepatitis (NASH) to cirrhosis and even hepatocellular carcinoma [1–3].

The pathogenesis of this disease has not been fully elucidated until now. In recent years, the “multiple parallel hits hypothesis” of NAFLD has attracted wide attention from researchers. In this hypothesis, a number of diverse parallel processes including adipose tissue-derived signals, gut barrier function, genetic factors, endoplasmic reticulum stress, and related signaling networks might contribute to the evolution of NAFLD.

Studies in the past emphasized adipose tissue-derived signals. Some factors could destroy the balance of lipometabolism between adipocytes and hepatocytes and finally cause NAFLD. But actually, such research results cannot explain the pathogenesis of NAFLD perfectly. Since 1998 when Marshall proposed the conception of “gut-liver axis,” combining gut and liver together, more and more researchers have paid close attention to the role of gut barrier function in the pathogenesis of NAFLD.

## 2. Gut-Liver Axis

The anatomy of the liver provides its close interaction with the gut where nutrients and the microbiome contribute to the maintenance of a healthy metabolism and liver. Gut-derived nutrients and other signals are delivered to the liver via the portal circulation that has several unique features. The slow blood flow in the liver sinusoids permits interactions between gut-derived substances and hepatocytes, other liver parenchymal cells, and liver immune cells; this is further promoted by the fenestrated endothelium in the sinusoids [4]. The liver, the largest immune organ, hosts the entire spectrum of immune cell repertoire and has a remarkable capacity to recruit and activate immune cells in response to gut-derived metabolic or pathogen-derived signals. The effects of gut microbiota in liver diseases have been a major interest in recent years. A recent study places the liver in the center of the intersections between the host and the gut commensal microbiota [5]. Interestingly, bile acid produced by the liver can also modulate the microbiome as some bacteria utilize bile acids [6]. The imbalance of gut-liver axis is increasingly recognized as a major factor in NAFLD.

## 3. Gut Barrier Function

The ability to control uptake across the mucosa and protect from harmful substances in the gut lumen is defined as gut barrier function. The intestinal barrier is a complex system serving two critical functions for the survival of the individual: first, it allows nutrient absorption and second, it defends the body from dangerous macromolecule penetration [7, 8]. It is composed of four major aspects: physical, chemical, biological, and immunological barriers. In detail, physical barrier includes mucous layer, intestinal epithelial cells, and the tight junctions located at the apical part of it. Chemical barrier includes gastric acid, digestive enzyme, and bile acid. Immunological barrier refers to lymphocytes and immunoglobulin A (Ig A). Biological barrier is composed of normal intestinal flora, the important environmental factor for the energy absorption and storage. The patterns of manifestation are various, such as flora shift, small intestinal bacterial overgrowth (SIBO), the alteration of tight junction, and gut permeability increasing.

## 4. Gut Physical Barrier Function and NAFLD

Several researches from both experimental animal models and human studies provide growing evidence that the progression of NAFLD is related with the impairment of gut physical barrier function. Gut permeability refers to the character that some molecular substance can get through the intestinal epithelium by simple diffusion. The increased permeability can be one of important manifestations of gut physical barrier function impairment. Patients with NAFLD had significantly increased gut permeability compared with healthy subjects. Importantly, the increased permeability appears to be caused by disruption of intercellular tight junctions in the intestine, which is thought to be the key factor of gut physical barrier function [9].

A research by Rahimi et al. [10] showed that, in Iran, the morbidity of celiac disease in the patients with NAFLD is significantly higher than in people without NAFLD. As celiac disease is a typical disease with incomplete tight junction, this evidence really gave good support to the relationship between gut physical barrier and NAFLD.

In normal condition, intact tight junction in intestine can prevent bacteria and toxin from getting through and prevent the occurring of intestinal flora shift. When tight junction is impaired, the intestinal permeability will increase. LPS, a component of the outer membrane of Gram-negative bacteria, will rush into portal system. Increased levels of LPS entering the liver have multiple biologic effects. First, LPS induces recruitment and activation of inflammatory cells and proinflammatory cytokine production. Second, LPS modulates hepatocyte functions and results in cholestasis [11]. Third, LPS and proinflammatory cytokines induce production of acute phase reactants by hepatocytes in the liver including serum amyloid A, LPS binding protein (LBP), fibrogen, C-reactive protein, IL-6, and ceruloplasmin [12]. It has been proposed that normal hepatocytes have a role in “detoxification” of the portal blood including elimination of LPS [13, 14]. Altered production of LBP, soluble CD14, and anti-LPS antibodies that all act by binding circulating LPS modulates the biologically active form of LPS that leads to inflammation.

## 5. Gut Chemical Barrier and NAFLD

Bile acid secreted by liver not only plays an important role in emulsifying fats and absorbing lipid-soluble vitamin [15] but also maintains the gut barrier function and homeostasis by inhibiting SIBO [16].

In mice model with fructose-induced NAFLD, the experimental group was fed with bile acids while control group was not. The markers of hepatic steatosis and portal endotoxin levels in the experimental group were markedly attenuated compared with control group. But the nuclear receptor of bile acids, farnesoid X receptor (FXR), and its mediated short heterodimer partner (SHP) were not significantly different between the two groups, which indicates that the reason why bile acids can relieve NAFLD may be the alteration of gut bacteria and endotoxin [17] besides through FXR-sterol response element-binding protein-1c (SREBP-1c) cascade signal transduction system to regulate hepatic triglyceride metabolism [18, 19].

Researches also show that some microbes can affect the metabolism of bile acids by synthesizing bile salt hydrolase, disturbing the signal path of lipid metabolism. As a result, it can induce the lipid peroxidation and, finally, hepatic lipid accumulation. Martin and his colleagues [20] transplanted infant intestinal flora into the gut of germ-free mice and found that conjugated bile acid in terminal ileum increased and plasma lipoprotein decreased, but hepatic triglyceride increased at the same time. They inferred that the alteration of gut flora can promote the bile acid enterohepatic circulation, inhibit the synthetizing and secretion of VLDL and LDL, and result in the hepatic steatosis at last [21].

## 6. Gut Immunological Barrier and NAFLD

Toll-like receptors (TLRs) are also expressed in the intestinal epithelium. As the critical molecules, the signal transduction of TLRs is related to the evolution of NAFLD. Wild-type (WT) mice fed high-fat (HF), fructose-rich, or methionine/choline-deficient (MCD) diet show severe steatosis or steatohepatitis. In contrast, TLR4 mutant mice on these diets have less steatosis or steatohepatitis, although LPS levels are equivalent to those in WT mice [22].

There are views that the products of the host cells destruction, namely, damage associated molecular patterns (DAMPs), were main ligand of TLRs [23]. They are mainly endogenous substance, such as free fatty acids (FFAs). It is FFAs that can stimulate the TLR2- or TLR4-dependent signal path directly. So this point emphasizes that FFAs are the key factor connecting fat intake in diet and TLR mediated disease [24].

However, such point was denied by Erridge and Samani [25]. They had done experiments of various kinds of cells, like macrophage, lipocyte, smooth muscle cell, endotheliocyte, and so forth, discovering that it is not FFAs that upregulate the expression of TLR stimulated gene products such as interleukin-1 (IL-1) and tumor necrosis factor-*α* (TNF-*α*), but it is intestinal bacterial structure or metabolic products that take apart in the TLRs signal transduction pathway. After this, there is more research carried out on their views, supporting that pathogen associated molecular patterns (PAMPs) from intestinal bacteria play a central role in the progress of NAFLD. Bacterial lipopeptide, LPS, and flagellin are recognized by TLR2, TLR4, and TLR5, respectively, while TLR3, TLR7, TLR8, and TLR9 are identified as the receptors which respond to bacterial nucleic acids. TLR2 generally forms heterodimers with TLR1 or TLR6. Specifically, the TLR2-TLR1 heterodimer recognizes triacylated lipopeptides from Gram-negative bacteria and mycoplasma, whereas the TLR2-TLR6 heterodimer recognizes diacylated lipopeptides from Gram-positive bacteria and mycoplasma. When PAMPs are recognized and bonded with corresponding TLRs, the activation of the transcription factor nuclear factor-kappa B (NF-*κ*B) and mitogen-activated protein kinases (MAPKs) occurs and proinflammatory genes such as inflammatory cytokines, adhesion molecule, and chemotactic cytokine are upregulated later on [26].

According to current opinion, such systematic, low level inflammatory response plays an important role in the pathogenesis of NAFLD [27, 28].

Besides TLRs, IgA secreted by gut is also an important part of gut immunological barrier function. IgA can inhibit pathogens from adhering to the mucous, thus taking effect in lumen [29]. Experiments confirmed that IgA had special affinity for gut Gram-negative* Bacillus*. 60%–80% of Gram-negative* Bacillus* are coated with IgA. When the intestinal mucosa is impaired, the quantity of secretory IgA (sIgA) plasmocytes and the Gram-negative bacteria coated with sIgA decreases; thus the small intestinal flora shift is promoted [30]. In this way, IgA is associated with the occurrence and development of NAFLD.

As we know, glutamine is the main material which can repair the intestinal epithelium. Supplement of glutamine can prevent the impairment of gut immunological barrier. Research has shown that, in the high-fat-induced NASH mice model, the blood transaminase and the hepatic inflammation scores of the treatment group with glutamine per os for 4 wk are significantly decreased compared with the control group [31].

## 7. Gut Biological Barrier and NAFLD

Data from mice experiments supported the idea that imbalance of intestinal flora was associated with NAFLD. The reasons why mice intestinal flora shift give rise to NAFLD are considered as follows.

1. Releasing LPS: it contributes to the development of the subclinical inflammatory state and insulin resistance associated with type 2 diabetes and obesity by stimulating the innate immune system and triggering the release of proinflammatory cytokines from adipose tissue [32]. This insulin resistance is associated with steatosis [33].
2. Increasing endogenous ethanol production [34]: Cope et al. demonstrated the critical role of intestinal flora in endogenous ethanol production and suggested that treatment of bacterial overgrowth might reduce potentially harmful levels of intestinally derived ethanol in humans with NAFLD. Indeed, a subsequent pilot study of patients with NASH demonstrated increased breath ethanol concentrations among obese females with this condition, confirming the suspicion that increased intestinal ethanol production occurs in some humans with NAFLD [35].
3. Reducing choline bioavailability in human body: deficiency of choline may result in the inability to synthesize phosphatidylcholine (PC) necessary for the assembly and secretion of very low-density lipoprotein (VLDL) and subsequent accumulation of triglyceride in liver [36]. Recently, some basic study has shed light on the view that gut flora can regulate energy metabolism [37, 38].

When intestinal flora homeostasis is disturbed, human energy metabolism is also changed accordingly. The mechanism through which gut microbiome regulates energy is considered as follows: (1) capability of breaking down otherwise indigestible alimentary polysaccharides, increasing the efficiency of energy metabolism, and providing more energy for the host [39], (2) gut microbiome-promoted storage of circulating triglycerides into adipocytes by suppressing intestinal secretion of an inhibitor of adipose tissue lipoprotein lipase called fasting-induced adipose factor (FIAF), also known as angiopoietin-like protein 4 [40], and (3) an increased activity of the enzyme AMP-activated protein kinase, which activates key enzymes of mitochondrial fatty acid oxidation, including acetyl-CoA carboxylase and carnitine palmitoyltransferase I. In this way, it plays a critical role in the pathogenesis of diabetes and obesity [41, 42].

At present, it is considered that above reasons cause the metabolic imbalance between adipocytes and hepatic cells.

Some research focuses on the role of diet in the gut biological barrier in recent years. In healthy volunteers, soluble PAMPs produced by inherent enteropermanent planting bacteria are quite little, only about 0.3 ng/mL, which indicates that PAMPs, the key factor closely associated with NAFLD, would probably come from diet [43]. The research result of Westerners' diet showed that, in unprocessed food, the level of PAMPs is too low to detect. However, in processed food, it is much higher than the average level in small intestine [44].

A human experiment by Spencer et al. [37] reported that each individual's microbiome remained distinct in short time, even though all subjects were fed identical diets in which choline levels were manipulated. Variations between subjects in levels of Gammaproteobacteria and Erysipelotrichi were directly associated with changes in steatosis in each subject. It may open avenues for further research and open vistas on looking for intestinal bacterial biomarkers which are associated with NAFLD.

Of course, NAFLD also can promote intestinal flora shift at the same time. Various inflammatory mediators were produced along with the progress of NAFLD. For example, IL-1 and interferon (IFN) can inhibit feeding center and cause anorexia and gastrointestinal hypomotility. Prostaglandin-2 (PGE-2) and platelet activating factor (PAF) can induce gastrointestinal dysfunction, decreased or lost migrating motor complex (MMC), and the stasis of intestinal contents and, as a result, the alteration of small intestinal flora occurs [45].

In several animal experiments, researchers fed animals with prebiotics and probiotics and found hepatic steatosis was relieved, the levels of aminotransferase reduced, and insulin resistance improved. These evidences strongly support that maintaining gut biological barrier function plays a critical role in the progress of NAFLD.

## 8. Conclusion

**Figure 1:**
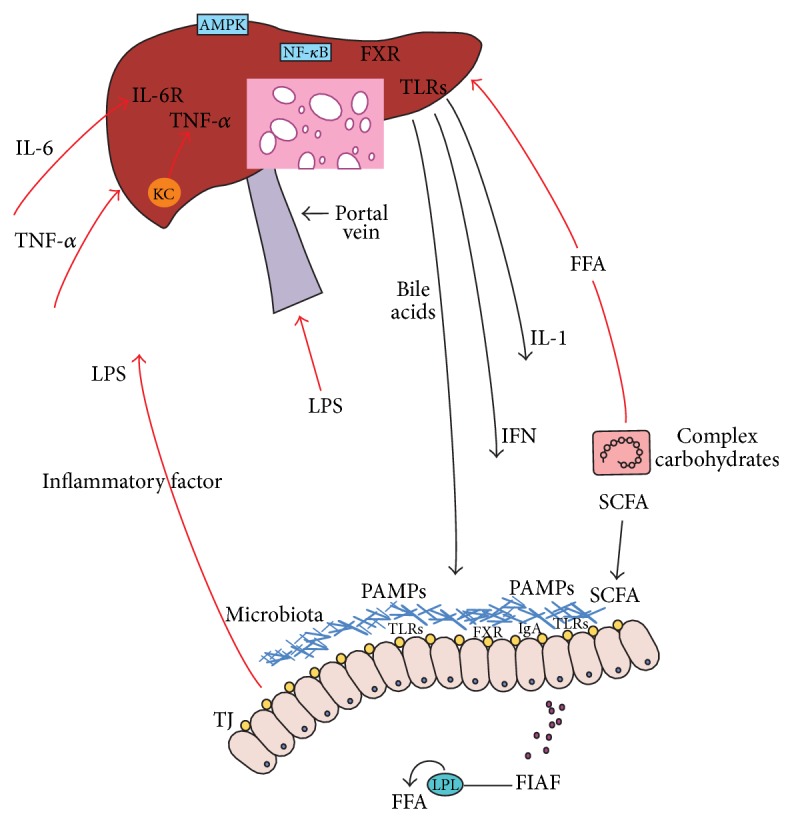
Gut-liver axis in NAFLD.

The four aspects of gut barrier function including physical, chemical, biological, and immunological barriers are closely related to each other and inseparable from NAFLD (Figure 1). For instance, intestinal epithelial cells are important part of physical barrier. When the permeability of physical barrier is increased, LPS will rush into portal system and induce the progress of NAFLD. At the same time, TLRs, the important member of innate immunity, are also expressed in the epithelium and their signal transduction is relevant with NAFLD. The ligand of TLRs is PAMPs from microbiome. The intestinal microbiota has a major role in shaping the host immune response and commensal bacteria shape the integrity of the gut mucosa [46]. The exchange of gut flora can lead to the abnormal accumulation of triglyceride in liver through inhibiting the synthesis and secretion of VLDL and LDL and finally cause NAFLD. IgA, a critical part of immunological barrier, works as the protector of gut mucosa through coating the Gram-negative bacilli and takes effect. The alteration of gut flora leads to the increasing of alcohol in lumen and destroys the intact of gut mucosa and physical barrier. No matter which aspect of gut barrier is destroyed, the other aspects will also be impaired and all of them combine together to cause the occurrence and development of NAFLD.

Of course, as previously mentioned, the impairment of gut barrier can lead to NAFLD, and vice versa NAFLD progressing to certain extent can also affect the gut barrier function. That is a vicious circle. If gut barrier (physical, biological, immunological, and chemical barrier) “forms” the first line of defense against the exogenous substances, liver can be the second one.

As for which aspect in the gut barrier plays the core role still needs further research to clarify. According to the existing research results, the exact relationship between form and extent of gut barrier impairment and the progress of NAFLD (NAFL, NASH, and associated liver cirrhosis) is still unclear and needs more research. An experiment by Miele et al. showed that, in the patients with NAFLD, gut permeability and SIBO are significantly positively associated with the severity of liver steatosis but not with inflammation. Gäbele and his colleagues [47] questioned this point with their new data. Application of dextran sulfate sodium (DSS) is a colitis model in mice characterized by damage of the intestinal barrier. They fed mice with high fat (HF) and DSS, setting NASH animal models with gut barrier impairment, and found that the hepatic inflammation was more severe in this group than in mice only fed with HF. HF + DSS mice also showed increased hepatic fibrosis. The result of Miele maybe owes to the lack of sample capacity, so this may require human experiments with a larger sample to confirm. Given that gut barrier function plays an important part in the pathogenesis of NAFLD, it is attractive to explore therapeutic interventions that could protect the gut barrier function. Due to the restrictions of ethics, there are only 10 experiments results about preventing NAFLD with probiotics and prebiotics published up to now [48]. Starting with maintaining gut barrier function and resetting healthy and balanced gut-liver relationship to treat NAFLD requires further studies to evaluate the effect.
